# Current Landscape of Micro-LED Display Industrialization

**DOI:** 10.3390/nano15090693

**Published:** 2025-05-04

**Authors:** Yang-En Wu, Chia-Hung Tsai, Li-Yin Chen, Fang-Chung Chen, Hao-Chung Kuo

**Affiliations:** 1 Department of Photonics, College of Electrical and Computer Engineering, National Yang Ming Chiao Tung University, Hsinchu 30010, Taiwan; ivanwu.ee12@nycu.edu.tw (Y.-E.W.); sppsai.ee12@nycu.edu.tw (C.-H.T.); fcchendop@nycu.edu.tw (F.-C.C.); 2 AUO Corporation, No. 1, Li-Hsin Rd. 2, Hsinchu 300094, Taiwan; 3 Center for Emergent Functional Matter Science, National Yang Ming Chiao Tung University, Hsinchu 30010, Taiwan; 4 Semiconductor Research Center, Hon Hai Research Institute, Taipei 11492, Taiwan

**Keywords:** Micro-LED, bonding materials, pulse-width modulation (PWM), pulse-amplitude modulation (PAM), color conversion, cost reduction, transparent display

## Abstract

Micro-LED display technology has emerged as a significant area of interest, with numerous research teams globally approaching it from various disciplines. Concurrently, several enterprises have initiated production or plan to invest in equipment manufacturing. However, the industry currently lacks standardized production processes for Micro-LED displays. This is largely due to major manufacturers adapting their equipment and material choices to suit their specific product applications. Nevertheless, advancements in recent years and developments within the supply chain reveal a gradual convergence of technology across the sector. This review paper aims to provide an investment and cost analysis perspective of the current industrial landscape of Micro-LED technology. It examines key aspects such as the selection of bonding materials, differences in driving modes, considerations for native RGB versus color conversion, strategies for cost optimization, market information and unique differentiation features of Micro-LED displays. To make this paper accessible to a broader audience, including those outside the electronics industry, key technical processes are described with clear explanations and the relevant context.

## 1. Introduction

In recent years, Micro-LED technology has emerged as a focal point of research in the field of display technologies, garnering considerable attention from both academia and industry [1,2,3,4,5,6]. Its significance transcends the theoretical exploration of component characteristics and material integrations, offering substantial potential for transformative industrial applications. The display industry has experienced a series of groundbreaking innovations over the past century, each reshaping the technological landscape. These milestones include the progression of cathode ray tube (CRT) technology from monochrome to color displays, the introduction of active-matrix liquid crystal display (AMLCD) technology five decades ago [7,8], and the efficiency improvements in organic light-emitting diode (OLED) materials achieved over the past 30 years [9]. More recently within the last decade, significant advancements in Micro-LED manufacturing techniques have been realized [1,10,11,12,13,14,15,16,17,18,19,20].

These technological advancements underscore the persistent evolution and renewal within the display industry. The pursuit of superior visual experiences, energy-efficient technologies, and the realization of “display everywhere” has been driven by the inherent advantages of Micro-LED components. Their tiny size, high luminous efficiency, and resilience to high current densities enable the development of a wide range of display applications, from ultra-high-resolution televisions and augmented/virtual reality (AR/VR) devices to wearable electronics, automotive displays, and large-scale outdoor signage. In the automotive sector, there is strong interest from brand customers in transparent displays, foldable screens, and exterior vehicle displays. For large-format displays, luxury TVs and public information displays (PID) have also inspired various application scenarios, fully leveraging the strengths of Micro-LED technology—such as high color saturation, high brightness, low power consumption, and high transparency.

As a result, major panel manufacturers—including AUO, BOE, CSOT, Innolux, LGD, Samsung, and Tienma—have dedicated R&D teams actively developing Micro-LED technologies. Prior to 2021, most Micro-LED displays showcased by these companies were under 10 inches. From 2022 to 2024, while some demonstrations reached sizes above 100 inches through tiling, the individual panels were still mostly around 12 inches. In 2025, AUO took the lead by demonstrating a 42-inch panel produced on a Generation 4.5 substrate, using larger substrates to continually reduce manufacturing costs and pave the way for broader adoption of Micro-LED displays.

**Figure 1:**
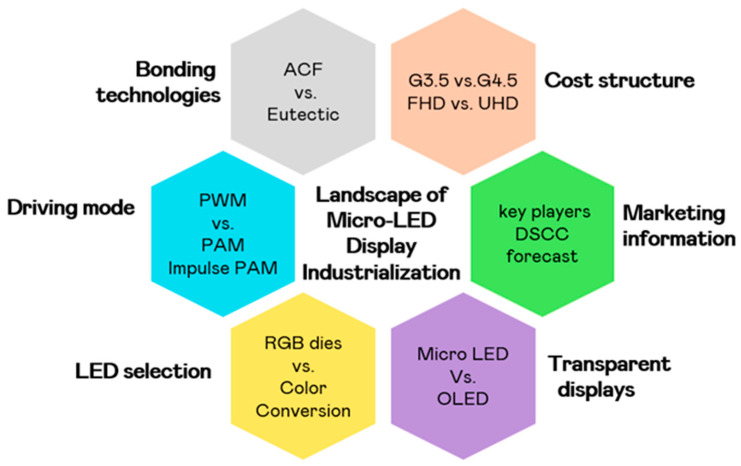
The current landscape of Micro-LED display industrialization. The topics are discussed in this study.

We have summarized the current landscape of Micro-LED display industrialization in Figure 1. Through these perspectives, this study seeks to elucidate the progress of industrializing this display fabrication technology. This review also presents a comprehensive overview of company investment strategies and product cost considerations that are shaping the industrialization of Micro-LED displays. It focuses on six key perspectives: bonding technologies, driving modes, LED selection, cost optimization, market information and transparent product applications. Together, these dimensions form a cohesive framework for understanding the fundamental logic and technological priorities driving Micro-LED development.

In addition to offering an in-depth analysis of Micro-LED industrialization, this review is designed to be accessible to readers from a range of technical backgrounds. While professionals in electronics and display technologies may benefit from detailed comparisons of the fabrication methods, we have also included contextual explanations to support readers from related fields such as materials science, optics, and applied physics. Clarity and background information are prioritized throughout to facilitate interdisciplinary understanding.

## 2. Selection of Bonding Materials for Micro-LED Displays

Bonding materials are used to attach the Micro-LED chips to the display substrate. This is a crucial step that determines the mechanical stability and electrical connectivity of the final display. For readers unfamiliar with this process, bonding is analogous to soldering in electronics assembly but on a micro-scale, where precision, thermal behavior, and optical performance are all critical.

**Figure 2:**
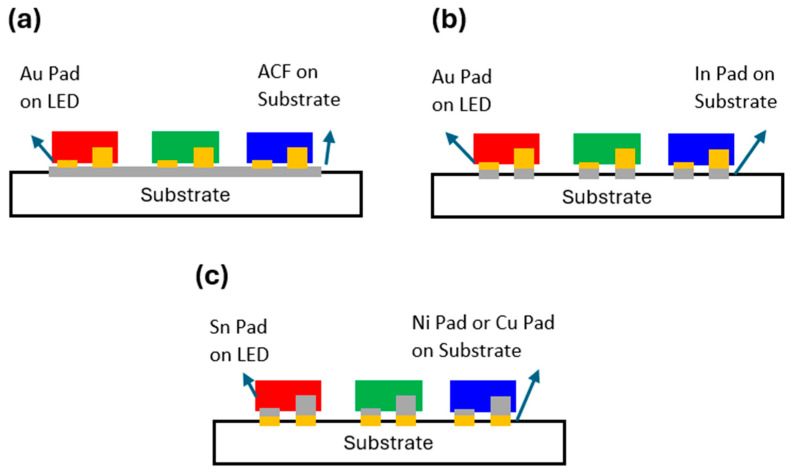
Schematics of different bonding materials: (**a**) Anisotropic conductive film (ACF) bonding. (**b**) Au/In eutectic bonding, and (**c**) Sn/Ni or Sn/Cu eutectic bonding.

The selection of bonding materials is critical for ensuring the secure and efficient attachment of Micro-LED components to glass substrates while maintaining high manufacturing yield and long-term reliability. The industry primarily employs three bonding approaches: anisotropic conductive film (ACF) bonding, Au/In eutectic bonding [21], and Sn/Ni or Sn/Cu eutectic bonding [22,23,24]. Each method offers distinct advantages and presents unique challenges in the context of Micro-LED industrialization. Figure 2 presents different Micro-LED bonding techniques along with their corresponding under bump metallization (UBM) structures, each tailored to specific packaging requirements. Figure 2a illustrates ACF bonding, where Au pads on the LED interface with an ACF substrate, provide both electrical connectivity and mechanical attachment. Figure 2b depicts Au/In eutectic bonding, in which Au pads on the LED are directly bonded to In (indium) pads on the substrate, ensuring a low-resistance electrical connection. Figure 2c shows Sn/Ni or Sn/Cu solder bonding, where Sn (tin) pads on the LED form eutectic or metallic bonds with Ni (nickel) or Cu (copper) pads on the substrate, enhancing both mechanical robustness and electrical conductivity. These bonding techniques significantly impact the electrical performance, reliability, and process compatibility of Micro-LED displays. Each method presents distinct advantages and challenges in the industrialization of Micro-LED technology, influencing factors such as manufacturing scalability, long-term stability, and overall production efficiency.

### 2.1. Anisotropic Conductive Film (ACF) Bonding

ACF bonding is a well-established method widely used in mass production due to its mature process parameters and extensive industrial adoption. One of its key advantages is the ability to connect a large number of pads simultaneously. Additionally, ACF bonding operates at lower temperatures, typically ranging from 110 °C to 180 °C, reducing the thermal stress on sensitive components. However, despite these benefits, its applicability to Micro-LED technology is increasingly being challenged by several key limitations as follows.

- Constraints on chip miniaturization: To avoid short circuits, the positive and negative connection pads (P-pad and N-pad) on the Micro-LED chip must be spaced apart by at least three times the size of the conductive particles used in the bonding film. This spacing is necessary to prevent unintended electrical connections during the bonding process, as the conductive balls may spread and merge when heated and compressed. Insufficient spacing increases the risk of short circuits, particularly in miniaturized flip-chip designs where pad dimensions are already constrained. As a result, achieving finer pitch designs with ACF bonding becomes increasingly challenging, limiting its suitability for high-density Micro-LED arrays.
- Limited rework ability: ACF bonding poses significant challenges in rework ability due to the properties of the adhesive and conductive particles. Once bonded, the adhesive layer establishes a durable connection, making it difficult to separate the components without risking damage to the Micro-LED or the substrate. In cases of defects, removing the adhesive requires extensive cleaning, as residual conductive particles may remain trapped in the bonding interface, potentially causing electrical instability. Moreover, the thermal and mechanical stress exerted during rework can degrade both the LED and the substrate, further complicating defect correction. These limitations lead to increased rework time and costs, ultimately reducing production efficiency.
- Transparency challenges: In transparent display applications, maintaining optical clarity is essential, as any obstruction can degrade image quality and visibility. ACF contains conductive particles and adhesive materials that can scatter or absorb light, leading to reduced transparency. This issue becomes particularly significant when ACF is applied over large areas, as it can introduce visible seams or haze, compromising display uniformity. To mitigate these effects, ACF bonding must be confined to small, localized areas, which adds complexity to the bonding process and restricts design flexibility. Consequently, ACF bonding is less suitable for high transparency display technologies, where maintaining high transmittance is critical.

### 2.2. Eutectic Bonding

Eutectic bonding has emerged as the dominant bonding technique in Micro-LED industrialization, leveraging two primary variants: Au/In eutectic bonding, one of the earliest approaches, and Sn/Ni or Sn/Cu eutectic bonding, which has been widely adopted from traditional LED displays to Micro-LED applications. The widespread adoption of eutectic bonding can be attributed to several reasons as follows.

- Established material and process maturity: Both chip-on-board (COB) and pack-age-on-board (POB) configurations have extensively utilized these materials. Manufacturers are well-acquainted with their characteristics, process parameters, and reliability conditions. Its long history in LED packaging means that manufacturers are familiar with its thermal behavior, bonding strength, and overall performance, allowing for seamless integration into Micro-LED production. Additionally, eutectic bonding provides excellent mechanical stability and electrical conductivity, making it particularly advantageous for high-performance Micro-LED displays where precise alignment and strong interconnections are required.
- Strong supply chain infrastructure: The materials required for eutectic bonding, such as gold (Au), indium (In), tin (Sn), nickel (Ni), and copper (Cu), are widely available and supported by a mature global supply chain. This well-established infrastructure ensures a stable and cost-effective supply of bonding materials, reducing production costs and minimizing disruptions in large-scale manufacturing. As the demand for Micro-LED displays continues to grow, the ability to source and scale eutectic bonding materials efficiently further strengthens its position as the preferred bonding technique in the industry.
- Superior rework ability: Unlike ACF bonding, which is difficult to rework once applied, eutectic bonding offers greater flexibility in defect correction and rework ability. If alignment issues or bonding defects occur during assembly, reflowing the eutectic material allows for debonding without significantly compromising device integrity. This capability is particularly important for Micro-LED applications, where high precision and yield are critical to mass production. The ability to efficiently repair defective units reduces overall material waste and enhances manufacturing efficiency, further driving the adoption of eutectic bonding in next-generation Micro-LED displays.

Recent advancements in eutectic bonding have demonstrated its scalability for smaller Micro-LED chips. As bonding materials continue to evolve, collaborative efforts across the supply chain aim to further enhance production efficiency, improve process compatibility, and reduce manufacturing costs. The convergence of these technological advancements underscores the increasing role of eutectic bonding in the future of Micro-LED display manufacturing.

## 3. Differences in Driving Modes for Micro-LED Displays

The driving mode of Micro-LED displays plays a crucial role in determining their power efficiency, color accuracy, and overall performance. These displays rely on active driving circuits built on thin-film transistor (TFT) substrates, which regulate the current supplied to individual Micro-LED elements within each pixel. The choice of driving method significantly impacts energy consumption, circuit complexity, and display uniformity, making it a key consideration in Micro-LED development.

**Figure 3:**
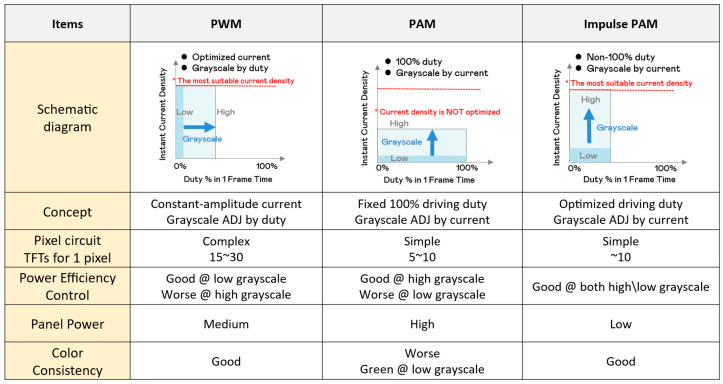
The comparison of PWM/PAM/impulse PAM driving modes.

Traditionally, pulse width modulation (PWM) has been widely used due to its high energy efficiency and ability to maintain color stability [25,26] As shown in Figure 3, PWM controls brightness by adjusting the duty cycle of a constant-amplitude current, ensuring that Micro-LEDs operate within their optimal efficiency range. However, as display resolutions increase and manufacturing challenges arise, the limitations of PWM—such as complex circuit design and scalability concerns—have become more apparent.

To address these issues, the industry has explored pulse amplitude modulation (PAM) as an alternative approach [27]. Unlike PWM, which regulates brightness by switching LEDs on and off, PAM adjusts the amplitude of the driving current, as shown in Figure 3. This method simplifies circuit design and improves manufacturability but introduces challenges related to efficiency loss at low grayscale levels and potential color inconsistencies.

Building on PAM, impulse PAM has emerged as a refined solution, combining the advantages of both PWM and PAM while mitigating their drawbacks. As illustrated in Figure 3, impulse PAM optimizes light emission timing by introducing shorter duty cycles, allowing Micro-LEDs to operate at peak efficiency while maintaining color stability. This approach not only enhances energy efficiency but also simplifies circuit complexity, making it a promising technique for next-generation Micro-LED displays. Figure 3 organizes and compares the differences among the three driving modes. The following sections provide a detailed analysis of PWM (Section 3.1) and PAM (Section 3.2), highlighting their advantages and limitations. Finally, Section 3.3 explores impulse PAM as an optimized driving method, highlighting its role in advancing Micro-LED technology.

### 3.1. PWM Driving

Pulse width modulation (PWM) has been widely adopted in Micro-LED displays due to its energy efficiency, color stability, and effective brightness control. PWM adjusts brightness by modulating the duty cycle of a constant-amplitude current, ensuring that Micro-LEDs operate within an optimal efficiency range across different grayscale levels.

#### 3.1.1. Advantages of PWM Driving

- Efficiency control: PWM maintains high energy efficiency by keeping the driving current within an optimal range across all grayscale levels. This helps reduce power consumption while maintaining stable brightness performance.
- Color consistency: One of the major advantages of PWM is its ability to minimize color shifts, particularly in green LEDs, which are highly sensitive to current variations. Because PWM maintains a constant current amplitude, color accuracy is preserved across different brightness levels.

#### 3.1.2. Challenges of PWM Driving

- Complex circuitry and manufacturing Yield Issues: PWM requires precise current regulation for each pixel, leading to a greater number of TFT components per pixel in active matrix driving circuits. This added circuit complexity increases production costs, impacts the manufacturing yield dramatically, and complicates large-scale fabrication. As display resolution increases, the number of required transistors grows, making PWM less scalable for high-density Micro-LED displays.
- Power efficiency at high brightness: While PWM is efficient at lower brightness levels, maintaining high brightness requires longer duty cycles, which can increase power consumption. This issue becomes more significant in large-area or high-resolution displays, where managing power distribution efficiently is critical.

### 3.2. PAM Driving

Pulse amplitude modulation (PAM) offers an alternative approach to Micro-LED driving by adjusting the amplitude of the driving current instead of modulating the duty cycle. Unlike PWM, which rapidly switches LEDs on and off, PAM varies the current level continuously, directly controlling brightness intensity.

#### 3.2.1. Advantages of PAM Driving

- Simplified circuit design: Since PAM does not require high-frequency switching, it reduces the number of transistors needed per pixel, leading to simpler circuit architecture and improved manufacturing yield. This makes PAM more scalable for high-resolution, high-pixel-density Micro-LED displays, where minimizing circuit complexity is crucial.
- Reduced power consumption at high brightness: PAM is more power-efficient at high brightness levels, as it does not rely on prolonged duty cycles like PWM. This makes it well-suited for applications requiring high brightness displays while maintaining energy efficiency.

#### 3.2.2. Challenges of PAM Driving

**Figure 4:**
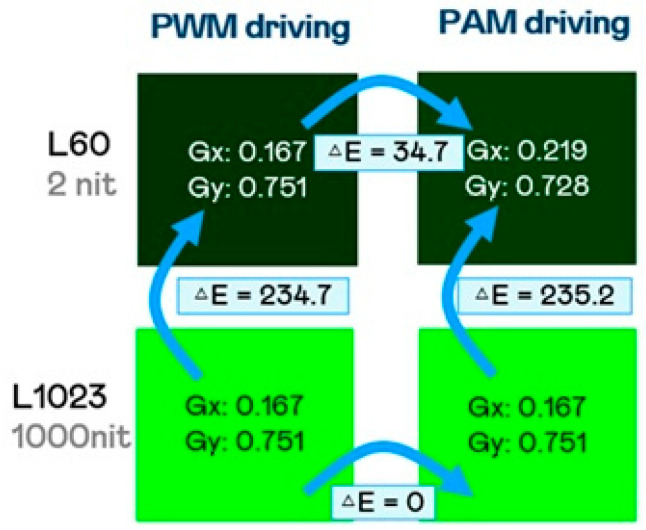
Color difference ΔE green color analysis between different grayscale and driving modes.

- Efficiency losses at low grayscale levels: Unlike PWM, where the driving current remains constant, PAM reduces current at lower brightness levels, leading to efficiency losses. At low grayscale levels, LEDs may operate below their optimal efficiency range, resulting in higher power dissipation and potential non-uniformity.
- Color inconsistencies: Since PAM directly modulates current, color shifts may occur, particularly in green LEDs, which are more sensitive to current variations. These color inconsistencies can become more pronounced at lower brightness levels, affecting display quality. We measured a 20 × 40 μm flip chip Micro-LED display and compared the green color differences in visual perception between PWM and PAM driving modes, analyzing the results using ΔE as in Figure 4. At high gray level L1023, approximately 1000 nits, the comparison showed ΔE = 0, indicating that the human eye can hardly discern any difference between the two driving modes. In contrast, at low gray level L60, around 2 nits, the comparison ΔE = 34.7. Since human perception of color is less sensitive at low gray levels, the slight color differences between the different driving methods are generally acceptable. When comparing high and low gray levels, the PWM driving modes showed ΔE = 234.7, while the PAM driving method showed ΔE = 235.2. The difference between the two is minimal, with the main contributing factor to ΔE being the variation in brightness, while the color differences can be largely disregarded.

Initially, concerns about power consumption and color shifts led some manufacturers to hesitate in adopting PAM for Micro-LEDs. However, further research and industry experience revealed that these issues were less severe than anticipated. As a result, manufacturers began shifting toward PAM-based approaches, prioritizing simplified circuit design and improved yield over the more complex architecture required for PWM.

### 3.3. Impulse PAM Driving

To further optimize Micro-LED performance, the industry has developed impulse pulse amplitude modulation (impulse PAM)—a refined approach that builds on traditional PAM while addressing its efficiency and color stability limitations. By introducing shorter duty cycles, impulse PAM allows Micro-LEDs to operate at peak efficiency while maintaining display uniformity, making it a promising solution for next-generation Micro-LED displays.

#### 3.3.1. Advantages of Impulse PAM Driving

- Impulse PAM works by flashing the LED in very short bursts instead of keeping it on continuously. This helps keep the LED operating in its most efficient range and reduces unnecessary heat generation. By precisely controlling the timing of light pulses, this technique enhances power efficiency and minimizes heat generation, similar to PWM, making it ideal for high-resolution and high-brightness Micro-LED applications. The duty cycle length can be further optimized based on specific product requirements, enabling greater design flexibility.
- Historical precedents in display technology: The concept of shortened duty cycles was successfully utilized in prior display technologies. Early cathode ray tube (CRT) displays employed similar techniques to improve visual clarity and reduce motion blur. However, liquid crystal displays (LCDs) were unable to adopt this approach due to their reliance on backlighting, where shorter duty cycles would negatively impact transparency and power consumption. By leveraging this principle, impulse PAM provides a proven, yet innovative solution tailored to self-emissive Micro-LED displays.

#### 3.3.2. Driving Mode Comparison Between PAM and Impulse PAM

**Figure 5:**
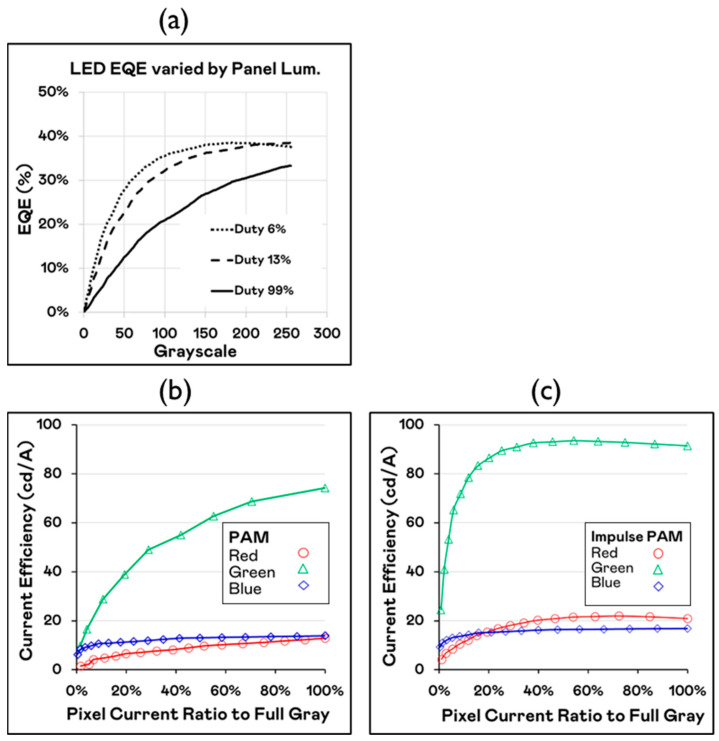
LED EQE for different grayscale and driving mode: (**a**) LED EQE tendency change as duty adjustment; (**b**) RGB chip current efficiency by PAM driving mode; (**c**) RGB chip current efficiency by impulse PAM driving mode.

We measured the 20 × 40 μm flip chip RGB Micro-LED die luminance versus driving current. Figure 5a compares three LED driving duties of 6%, 13%, and 99%. It can be observed that as the duty cycle increases, the EQE (external quantum efficiency) decreases. Therefore, in panel design, it is essential to consider the optimal driving duty to optimize efficiency. This also indicates that the panel temperature under impulse PAM (and also PWM) is lower than that of traditional PAM driving methods. Figure 5b indicates the current efficiency of RGB LED chips at various gray levels under PAM driving mode. Figure 5c shows that by changing to impulse PAM driving, it is possible to set a suitable duty cycle to achieve better efficiency.

#### 3.3.3. Industry Transition to Impulse PAM

The evolution of driving modes in Micro-LED displays reflects an ongoing effort to balance technical performance with practical manufacturability. While PWM driving offers strong efficiency and color stability, its complex circuit design and its impact on production yield limit its scalability. Similarly, traditional PAM driving, while simplifying circuit architecture, introduces challenges such as efficiency loss at low grayscale levels and color uniformity issues.

The transition to impulse PAM represents a pragmatic industry shift, combining the efficiency of PWM with the simplified circuit architecture of PAM. By optimizing light emission timing and reducing unnecessary power consumption, impulse PAM improves manufacturability, enhances display quality, and enables more efficient Micro-LED performance. This advancement underscores the industry’s focus on developing scalable, high-efficiency driving methods to accelerate the adoption of next-generation Micro-LED displays.

## 4. Considerations for Native RGB and Color Conversion Technologies

Choosing between native RGB Micro-LEDs and using a single color LED with color-converting materials is one of the central decisions in display design [28,29,30,31]. For general readers, this is similar to using separate colored lights (RGB) versus using a white light and putting colored filters on top. Each approach has trade-offs in terms of efficiency, manufacturability, and repairability. Historically, the mass production and efficiency of red LEDs have posed significant obstacles in the industry. A decade ago, very few manufacturers could produce high-quality red LEDs, and even fewer were capable of developing effective red Micro-LEDs. In addition to manufacturing challenges, red LEDs suffer from significant efficiency degradation at elevated temperatures, further complicating their production and integration.

To address these challenges, color conversion technology has emerged as a potential solution, leveraging blue Micro-LEDs as a single light source while using phosphor-based or quantum dot (QD)-based conversion materials to generate red and green emission. However, despite the theoretical advantages of color conversion, recent advancements in red Micro-LED technology have allowed more manufacturers to produce high performance red Micro-LEDs, although the issue of thermal efficiency loss remains unresolved.

**Figure 6:**
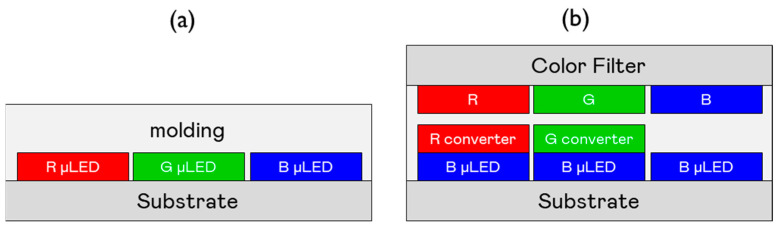
Schematics of RGB and color conversion structures: (**a**) Native RGB Micro-LED configuration; (**b**) Blue Micro-LED combined with color conversion materials.

Figure 6a illustrates a typical native RGB configuration, which employs separate red, green, and blue Micro-LED chips, each directly emitting its respective color. In contrast, Figure 6b depicts a color conversion structure, where blue Micro-LEDs serve as the primary light source, and color conversion materials generate red and green emissions. A diffuser is incorporated to enhance color mixing, while a color filter (CF) substrate is positioned on top to improve color accuracy. Although color conversion technology simplifies manufacturing by using a single LED type, RGB dies remain the preferred solution due to their higher efficiency, more advanced mass transfer processes, and broader applicability across various display technologies.

Despite the theoretical advantages of color conversion, RGB die production continues to lead the industry, driven by several key factors, which are discussed in the following sections.

### 4.1. Maturation of RGB Mass Transfer Technology

The mass transfer process for RGB Micro-LEDs has advanced significantly, reducing the complexity gap between three-color (RGB) and monochrome chip transfer. Today, the difficulty of transferring RGB Micro-LEDs is comparable to that of single-color chips, leading to greater industry acceptance of RGB solutions. In contrast, integrating color conversion technology introduces additional process complexity and requires specialized manufacturing equipment, resulting in higher production costs. These added burdens increase investment requirements without delivering substantial improvements in product value, making RGB dies a more commercially viable option for large-scale manufacturing.

### 4.2. Inefficiencies in Color Conversion Materials

Despite advancements, color conversion materials, particularly those used for blue-to-red and blue-to-green emission, remain significantly less efficient than native RGB dies. As of our measurement of about 20 × 40 μm Micro-LED, the current luminous efficiency of red Micro-LED dies is approximately 21 cd/A, and this can be enhanced by about 20% through advancements in manufacturing processes, with the aim of reaching 25 cd/A by the end of 2025. In contrast, when using color conversion technology to transform blue light into red, the efficiency decreases to around 14 cd/A. Furthermore, green Micro-LED dies exhibit a luminous efficiency of about 100 cd/A; however, when color conversion technology is applied to convert blue light into green, this efficiency is reduced to just 50 cd/A. This inefficiency stems from energy losses during the conversion process, which reduces overall luminous efficiency and limits their suitability for high-performance displays. As a result, color conversion-based products struggle to achieve the same brightness and power efficiency as native RGB solutions.

In addition to efficiency concerns, environmental regulations further restrict the use of high-performance color conversion materials. RoHS compliance prohibits cadmium-based (Cd-containing) quantum dots, which offer superior conversion efficiency. The available Cd-free InP-based [32,33] or perovskite QD [34] alternatives have lower performance, making it difficult to achieve the color purity and brightness required for high-quality Micro-LED displays.

Due to these technical and regulatory limitations, color conversion technology remains in the developmental stage, preventing its widespread adoption in mass production.

### 4.3. Broader Applicability of RGB Components

From an application perspective, RGB dies offer greater versatility across a broad range of display technologies, from low-resolution to ultra-high-resolution displays. This advantage is particularly pronounced in transparent displays, where manufacturers prioritize high aperture ratios to maximize transparency.

Additionally, considering the structure of the display module, employing native RGB micro-LED technology can enhance luminous efficiency by increasing the simple molding structure around the LED dies, such as using black and white glue. Both optical simulations and actual experimental results indicate that this approach can improve luminous efficiency by 20% [35]. Furthermore, the micro-lens array is used to optimize the LED pixels light output angle to reduce color mixing problems between adjacent LED pixels. At the same time the micro-lens can also improve the efficiency of LED light coupling, thereby further improving the LED light output efficiency [36]. Conversely, color conversion technology requires additional process space and involves more complex design considerations, creating bottlenecks in production scalability. Additionally, manufacturers must develop and maintain two separate technology platforms if both RGB and color conversion-based displays are pursued in parallel, further complicating adoption. These challenges reinforce RGB dies as the preferred solution for large-scale manufacturing.

### 4.4. Temperature Compensation for RGB Components

**Figure 7:**
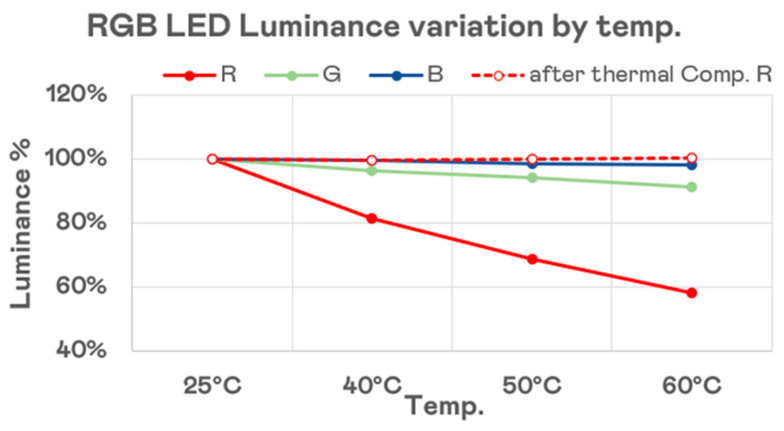
RGB LED luminance variation by temperature.

While RGB dies and color conversion materials respond differently to temperature changes, temperature feedback mechanisms have been developed to compensate for color shifts in RGB LEDs at high temperatures. Advanced thermal management systems allow real-time adjustments, reducing wavelength shifts and minimizing variations in luminous efficiency. Additionally, engineering optimizations have significantly reduced temperature-induced discrepancies, ensuring that any perceptible differences between RGB dies and color conversion technology remain minimal in practical applications. These advancements further reinforce RGB technology as the dominant choice for Micro-LED display production. Figure 7 illustrates the luminance degradation trend of 20 × 40 μm RGB Micro-LEDs as we measured the temperature changes from 25 °C to 60 °C. It can be observed that the red LED experiences the most significant degradation, dropping to only 60%. This issue can be addressed through thermal compensation technology, as indicated by the red dashed line, which shows that luminance can be kept to 100%.

### 4.5. Industry Trade-Offs and Future Outlook

The industry continues to evaluate the trade-offs between native RGB dies and color conversion technology. While color conversion offers a potential alternative to address red Micro-LED efficiency challenges, it remains limited by technical, commercial, and environmental constraints. On the other hand, RGB dies—supported by mature mass transfer techniques, higher energy efficiency, and broad application compatibility—continue to lead Micro-LED display manufacturing. Additionally, thermal compensation solutions have further mitigated the historical disadvantages of RGB dies, reinforcing their position as the primary technology for high-performance Micro-LED displays.

As technological advancements progress in both approaches, RGB dies are expected to remain the mainstream solution, while color conversion technology may serve as a complementary alternative in specific applications.

## 5. Directions for Cost Optimization and Market Landscape in Micro-LED Displays

As high production costs continue to hinder the mass adoption of Micro-LED displays, manufacturers are increasingly investing in cost optimization strategies to improve scalability and market competitiveness. The cost structure of Micro-LED displays can be broadly categorized into two key aspects as follows.

### 5.1. Large-Scale Manufacturing Processes

The manufacturing process of Micro-LED displays consists of several stages, including TFT glass fabrication, mass transfer, and final packaging. Since this process shares similarities with LCD and OLED manufacturing, Micro-LED manufacturers can apply proven cost control strategies from these industries to improve efficiency.

**Figure 8:**
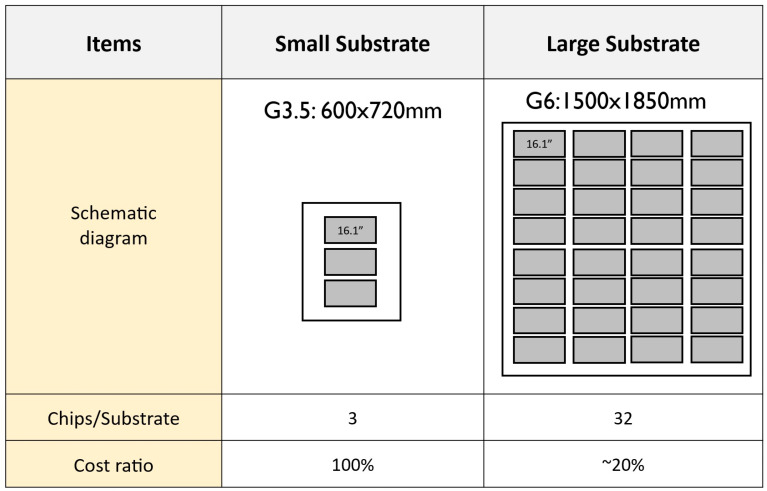
The TFT glass backplane cost ratio comparison between small substrate (G3.5) and large substrate (G6).

Figure 8 illustrates the cost impact of TFT glass size, also known as display generation, emphasizing its role as a key factor in display manufacturing costs. The figure compares the utilization of G3.5 and G6 TFT glass substrate. There are only three panels (3ea) that fit within one G3.5 glass substrate, resulting in lower panel yield per substrate and higher material costs per unit. There are thirty-two panels (32ea) that fit within one G6 glass substrate, increasing production output and optimizing glass utilization. We estimate that by using a G6 substrate design, the cost of the TFT backplane for each panel will be less than 50% of that required for a G3.5 substrate.

This comparison suggests that larger panels improve material efficiency, potentially leading to significant cost advantages in mass production.

### 5.2. Pixel-Based Cost Structure

**Figure 9:**
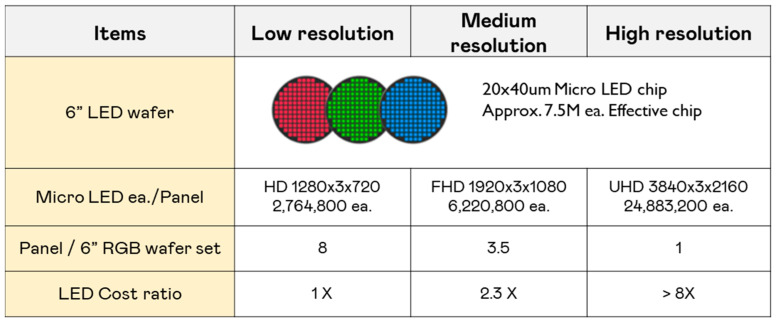
The estimated Micro-LED cost ratio vs. display resolution.

Unlike conventional displays where the cost mainly scales with the screen size, Micro-LED displays are more sensitive to how many individual pixels (tiny LEDs) that need to be placed and connected. Each pixel requires a separate Micro-LED chip, which makes high-resolution displays significantly more expensive to manufacture. Figure 9 illustrates how resolution directly impacts manufacturing costs in Micro-LED displays. In a low-resolution HD panel (1280 × 720), approximately 2.76 million Micro-LEDs are required, resulting in lower production costs due to the smaller number of LEDs per panel. In a high-resolution UHD panel (3840 × 2160), the number of Micro-LEDs increases exponentially to 24.88 million per panel, significantly raising production complexity, yield challenges, and overall costs. For example, if a 6” LED wafer is designed using a 20 × 40 μm Micro-LED chip, it is estimated that approximately 7.5 million effective chips could be produced. This indicates that each set of RGB LED wafers could provide around 8 HD-resolution panels, about 3.5 FHD panels, and 1 UHD panel. In other words, when only considering the cost of Micro-LED chips, the expense for UHD panels is roughly eight times that of HD panels.

As resolution increases, higher precision is required for LED placement, transfer accuracy, and yield efficiency, making resolution one of the most significant cost factors in Micro-LED display production.

### 5.3. Micro-LED Display Market Landscape

Over the past 30 years, cost optimization in display manufacturing has largely depended on increasing substrate sizes, which enhances production efficiency and reduces operational costs. However, maintaining acceptable yield rates remains critical to fully capitalizing on the benefits of large-scale manufacturing.

Recent advancements in the Micro-LED industry reflect this shift toward larger substrates. The successful industrialization of Micro-LED displays is closely linked to evolving market dynamics, key industry players, and emerging production trends. Leading panel manufacturers—such as AUO, Samsung, BOE, LG Display, Innolux, and Tianma—have actively invested in R&D, with several already demonstrating pilot production lines. The main challenge lies in deciding on the technology, as it directly affects investment costs. This includes the bonding materials, driving modes, and whether to adopt native RGB or color conversion, as discussed in this paper. In contrast, mass transfer technology is already relatively mature in the market. Many of these companies have also announced plans to scale up their manufacturing capabilities. Tianma and Innolux have adopted G3.5 substrates for mass transfer. AUO has revealed plans to begin production on G4.5 substrates by 2025, marking the largest substrate size used in Micro-LED manufacturing to date.

This transition, from substrates just a few inches in size to those measuring up to 730 × 920 mm, represents a significant technological milestone. Larger substrates offer substantial cost savings by reducing equipment investment, operational costs, and material waste, making them a key enabler for the mass adoption of Micro-LED displays.

In terms of application outlook, market research from DSCC (Display Supply Chain Consultants) projects significant revenue growth across several sectors, including TVs, wearables, AR/VR, and automotive/industrial displays. As shown in the DSCC Report [37], revenue is expected to surpass USD 700 million by 2028, with TV and AR/VR leading the adoption curve. While DSCC recently revised its 2027 forecast downward by USD 1 billion due to supply chain and cost challenges, the overall trend remains optimistic. This projection underscores the importance of aligning technological development with scalable production infrastructure and supply chain readiness. Companies that can successfully address yield, cost, and integration challenges are likely to lead the market in the coming years.

## 6. Comparison with Non-Transparent Self-Emissive Display Technologies

Micro-LED displays are being developed for various applications, including smartwatches, automotive displays, luxury televisions, and public information displays (PID). While they offer significant advantages in brightness, efficiency, and durability, their most distinctive and promising area of expansion lies in transparent displays. The following sections outline the key factors that set them apart.

### 6.1. Non-Transparent Self-Emissive Display Technologies Comparison

Conventional self-emissive displays adopt different manufacturing processes depending on their size. Displays smaller than 20 inches typically use fine metal mask (FMM) OLED technology, which allows for higher pixel density and precision. Displays ranging from 20 to 100 inches commonly rely on white OLED technology, which simplifies large-area fabrication while maintaining good color performance. For displays larger than 100 inches, tiling LED screen technology is the preferred choice, as it offers scalability and durability suitable for large-format applications.

Although Micro-LED displays outperform these existing technologies in panel specifications and overall performance, they are still in the early stages of commercialization. Their cost structure differs significantly from mass-produced display technologies, making them less competitive for mainstream applications in the short term. As a result, current Micro-LED production is primarily targeted at niche markets that demand custom specifications and high-performance displays. Despite low production volumes, these early-stage applications serve as a foundation for developing supply chains, enhancing process efficiency, and ultimately driving cost reductions that will support broader market adoption.

### 6.2. Advantages of Transparent Micro-LED Displays

Among transparent display technologies, white OLED currently dominates the market [38,39,40,41,42]. However, its limitations in brightness and lifespan restrict its broader application. In contrast, Micro-LED displays offer several advantages, making them a more promising alternative. White OLED struggles to achieve high brightness levels due to lifespan degradation at high luminance, whereas Micro-LED displays can easily exceed 1000 nits while maintaining long-term stability. Additionally, Micro-LEDs provide a half-brightness viewing angle of over 75 degrees, ensuring better visibility across different angles compared to white OLED.

Transparency is another key advantage of Micro-LED displays. White OLED panels require a larger emission area to lower current density and extend lifespan, which inherently reduces transparency. In contrast, Micro-LED components are much smaller and can withstand high currents, allowing most of the display area to remain transparent while using minimal space for light emission. This design significantly enhances transparency and makes Micro-LEDs well-suited for applications where clear visibility is essential.

**Figure 10:**
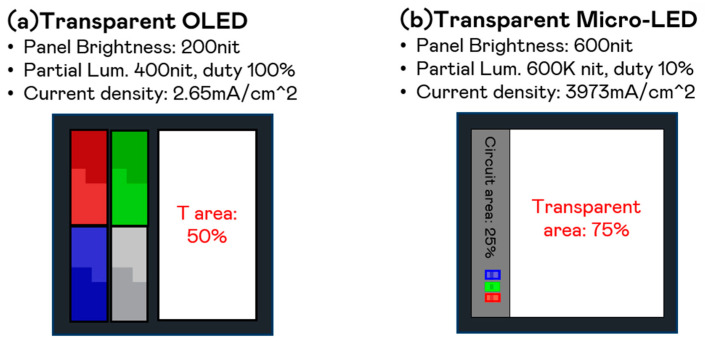
Schematics of (**a**) top-emission transparent OLED vs. (**b**) top-emission transparent Micro-LED.

Figure 10 compares a typical top-emission transparent OLED panel and a top-emission transparent Micro-LED panel, highlighting differences in brightness, emission area, transparency, and current density. Figure 10a represents a transparent OLED panel with a brightness of 200 nits, an emission area covering 50%, a circuit area of 25%, and a transparent area of 50%. It operates at a partial luminance of 400 nits with a 100% duty cycle and a current density of 2.65 mA/cm^2^. Figure 10b represents a transparent Micro-LED panel, achieving a higher brightness of 600 nits while significantly reducing the emission area to just 1%. The circuit area remains 25%, while the transparent area increases to 75%. This design results in an extremely high partial luminance of 600,000 nits but operates at a 10% duty cycle, leading to a current density of 3973 mA/cm^2^. While Figure 10b demonstrates higher brightness and greater transparency, it comes at the cost of a significantly higher current density, which may impact device reliability and longevity. This comparison highlights the trade-offs between emission area, brightness, transparency, and power efficiency in transparent display technologies.

Beyond brightness and transparency, Micro-LED displays also offer significantly lower power consumption compared to white OLED displays, with further potential for improving light efficiency. White OLED is constrained by efficiency losses in its color conversion and color-filtering processes, which limit color saturation and overall performance. In contrast, Micro-LED displays deliver higher color saturation and superior light efficiency, making them a more promising solution for transparent display applications.

## 7. Conclusions: The Industrialization and Future of Micro-LED Displays

Micro-LED displays have emerged as a next generation display technology, offering unmatched brightness, transparency, power efficiency, and superior visual performance. Throughout this review, we examined the key advancements and challenges shaping the industrialization of Micro-LEDs, spanning bonding material selection, driving mode optimization, RGB versus color conversion technologies, cost reduction strategies, and differentiation in transparent displays. These items have a significant impact on the company’s equipment investment and yield-related costs, and they are also the key concerns within the industry.

First, significant advancements have been achieved in bonding technologies, particularly in eutectic bonding, which has improved manufacturing yield, the extent of repair equipment investment, and reliability. Eutectic bonding technology, with its rework capabilities, is gradually replacing ACF bonding technology.

Second, the PAM driving mode streamlines circuit design, effectively addressing the complexities associated with PWM circuits and the challenges of very low production yields. To further enhance efficiency, especially at low grayscale levels—which often do not operate at the optimal current for LEDs—the adoption of impulse PAM can simultaneously improve both efficiency and color deviation issues. Impulse PAM has emerged as a refined driving method, offering a balance between efficiency, power consumption, and manufacturability.

Third, the choice between native RGB dies and color conversion technology directly influences whether additional equipment is required. While color conversion offers a single blue emitter, it suffers from red and green conversion efficiency loss. Furthermore, the requirement for an additional CF substrate complicates repairs and adds to costs. In contrast, native RGB LED technology has implemented thermal compensation techniques to tackle the high-temperature efficiency degradation of red LEDs. In addition to enhancing chip efficiency, advancements have been made in improving the overall luminous efficiency of display modules through enhanced molding structures. These advantages continue to make RGB dies the mainstream choice in high-performance and large-format applications.

Fourth, the cost structure of Micro-LED displays, which is heavily influenced by pixel resolution and substrate utilization, remains a barrier to mass adoption. Strategies such as increasing wafer usage, scaling up substrate generation sizes (e.g., G3.5 to G4.5), and adopting efficient transfer and bonding techniques are proving essential in reducing per-panel costs and improving manufacturability.

Fifth, transparent Micro-LED displays represent one of the most promising application frontiers. Compared to OLED-based transparent panels, Micro-LEDs achieve higher brightness, wider viewing angles, and better transparency through minimized emission area and robust current handling. However, ongoing efforts are required to address challenges such as high current density and thermal stability in such designs.

This review aims to serve not only as a technical reference but also as an accessible guide for professionals across disciplines—including materials science, optics, applied physics, and electronics—who are navigating the evolving landscape of Micro-LED technology. As the industry refines supply chains, fabrication processes, and material efficiencies, Micro-LED displays are moving closer to large-scale commercialization. While challenges in cost structure and process scalability persist, continued technological advancements are positioning Micro-LEDs as a transformative display solution capable of revolutionizing consumer electronics, automotive displays, large-scale signage, and transparent displays. With ongoing innovation, Micro-LED technology is poised to redefine display standards and drive the future of high-performance, energy-efficient visual systems.

## Data Availability

The data presented in this study are available from the corresponding author upon reasonable request.
